# Chromosome 20p11.2 deletions cause congenital hyperinsulinism via the loss of *FOXA2* or its regulatory elements

**DOI:** 10.1038/s41431-024-01593-z

**Published:** 2024-04-11

**Authors:** Thomas W. Laver, Matthew N. Wakeling, Richard C. Caswell, Benjamin Bunce, Daphne Yau, Jonna M. E. Männistö, Jayne A. L. Houghton, Jasmin J. Hopkins, Michael N. Weedon, Vrinda Saraff, Melanie Kershaw, Engela M. Honey, Nuala Murphy, Dinesh Giri, Stuart Nath, Ana Tangari Saredo, Indraneel Banerjee, Khalid Hussain, Nick D. L. Owens, Sarah E. Flanagan

**Affiliations:** 1 Department of Clinical and Biomedical Science, University of Exeter Medical School, Exeter, UK; 2 The Genomics Laboratory, Royal Devon University Healthcare NHS Foundation Trust, Exeter, UK; 3 Department of Paediatric Endocrinology, Royal Manchester Children’s Hospital, Manchester, UK; 4 Department of Health Sciences, School of Medicine, University of Eastern Finland, Kuopio, Finland; 5 Department of Paediatric Endocrinology and Diabetes, Birmingham Women’s and Children’s Hospital, Birmingham, UK; 6 Department of Biochemistry, Genetics and Microbiology, University of Pretoria, Pretoria, South Africa; 7 Department of Paediatric Endocrinology, Children’s University Hospital, Dublin, Ireland; 8 Department of Paediatric Endocrinology, Bristol Royal Hospital for Children, Bristol, UK; 9 Royal Cornwall Hospital, Truro, UK; 10 Division of Endocrinology, Sanatorio Güemes, Buenos Aires, Argentina; 11 Department of Paediatrics, Division of Endocrinology, Sidra Medicine, Doha, Qatar

**Keywords:** Endocrine system and metabolic diseases, Medical genetics, Epigenomics

## Abstract

Persistent congenital hyperinsulinism (HI) is a rare genetically heterogeneous condition characterised by dysregulated insulin secretion leading to life-threatening hypoglycaemia. For up to 50% of affected individuals screening of the known HI genes does not identify a disease-causing variant. Large deletions have previously been used to identify novel regulatory regions causing HI. Here, we used genome sequencing to search for novel large (>1 Mb) deletions in 180 probands with HI of unknown cause and replicated our findings in a large cohort of 883 genetically unsolved individuals with HI using off-target copy number variant calling from targeted gene panels. We identified overlapping heterozygous deletions in five individuals (range 3–8 Mb) spanning chromosome 20p11.2. The pancreatic beta-cell transcription factor gene, *FOXA2*, a known cause of HI was deleted in two of the five individuals. In the remaining three, we found a minimal deleted region of 2.4 Mb adjacent to *FOXA2* that encompasses multiple non-coding regulatory elements that are in conformational contact with *FOXA2*. Our data suggests that the deletions in these three children may cause disease through the dysregulation of *FOXA2* expression. These findings provide new insights into the regulation of *FOXA2* in the beta-cell and confirm an aetiological role for chromosome 20p11.2 deletions in syndromic HI.

## Introduction

Deletions that affect large regions of genomic DNA have an important role in both rare and common diseases [1, 2]. Monogenic disease can result when a deletion removes all, or part of the coding sequence of a single disease-causing gene. For example, recessively acting whole and/or partial gene deletions of *ABCC8* or *HADH* cause isolated persistent congenital hyperinsulinism (HI) [3–5], a genetically heterogenous condition characterised by severe hypoglycaemia due to inappropriate insulin secretion [6].

Some deletions that affect multiple genes can cause syndromic disease, with the extent of the deletion impacting on the clinical presentation [7]. The phenotype resulting from these large deletions can be readily explained when the deletion disrupts known monogenic disease genes, for example a deletion on chromosome 11p15.1 causes HI, enteropathy and deafness with loss of the *ABCC8* gene responsible for the HI and loss of the adjacent gene, *USH1C*, causing the enteropathy and deafness [8]. In other large deletion syndromes where HI is a rare feature [9], for example partial or full monosomy of the X chromosome causing Turner syndrome, and the 9p deletion syndrome, the precise genetic mechanism leading to the HI has not been fully determined [10].

Large deletions can cause disease through haploinsufficiency, by unmasking a recessive pathogenic variant on the opposite allele, by affecting a differentially methylated imprinted control region (e.g. Beckwith-Wiedemann syndrome [11]) or by disrupting a non-coding regulatory element that is critical for controlling gene expression. The latter is exemplified in a recent study where genome sequencing identified large (~4.5 kb) overlapping de novo deletions within an intronic region of the *HK1* gene in two children with HI. These deletions led to the discovery of a ~ 42 bp region that is critical for controlling HK1 expression within the insulin-producing pancreatic beta-cell [12].

For HI, large cohort studies have shown that routine genetic testing identifies a pathogenic variant in a known disease gene in 45–79% of cases [13, 14]. These pick-up rates are set to increase over time as more genetic causes of HI, such as *HK1*, continue to be discovered [12]. In this study we aimed to search for new genetic causes of HI in genetically unsolved individuals. We focussed on large deletions (>1 Mb) given their known contribution to the aetiology of this condition. We identified overlapping heterozygous deletions on chromosome 20p11.2 in five individuals (range 3–8 Mb); in two this included the HI gene, *FOXA2*. In the remaining three, the deletion encompassed multiple non-coding regulatory elements that are in conformational contact with *FOXA2* suggesting that they may cause disease by disrupting the regulation of *FOXA2* expression within the pancreatic beta-cell.

## Materials and methods

We studied an international cohort of 1063 individuals referred for routine genetic testing for HI. Clinical information was provided at referral using a standardised request form. Follow-up data by case note review were requested for individuals when a large deletion was identified. Informed consent was obtained from each of the parents/carers. This study was approved by the North Wales Research Ethics Committee (517/WA/0327).

### Routine screening of genes known to cause HI

DNA was extracted from peripheral blood leucocytes using standard procedures. Disease-causing variants in at least 12 known HI genes (*ABCC8*, *CACNA1D*, *GCK*, *GLUD1*, *HADH*, *HNF1A*, *HNF4A*, *INSR*, *KCNJ11*, *PMM2*, *SLC16A1* and *TRMT10A*) were excluded by targeted next generation sequencing in all 1,063 individuals as described previously [15]. This analysis generated an average of three million reads per sample. In all individuals routine screening by read depth analysis using ExomeDepth [16] excluded partial/whole gene deletions of the targeted genes. Deletions on chromosome X and 9p24, which are reported to cause HI [9], were also excluded using off-target reads [17].

### Searching for large deletions using whole genome sequencing

We initially searched for large deletions (>1 Mb) in 180 individuals using whole genome sequencing. Reads were aligned to the GRCh37/hg19 human reference genome with BWA mem (v0.7.15) followed by local re-alignment using GATK IndelRealigner (v3.7.0). Deletions were called by read depth analysis using SavvyCNV [17] (default parameters, bin size 2kbp, samples segregated by sequencing machine and sex). We searched for overlapping deletions present in at least three individuals with HI. Deletions which appeared in 882 in-house controls were assumed to reflect common variation or an artefact of the screening method and were excluded. Sequencing data was used to fine map the deletion breakpoints with the boundaries determined by manual inspection in the Integrative Genomics Viewer (IGV) based on the boundary of the drop in coverage.

### Replication screening studies using off-target next generation sequencing data

When a deletion was identified in three or more individuals, we used SavvyCNV [17] (transition probability 0.001, bin size 200 kbp, samples segregated by targeting panel and sex) to screen for overlapping deletions in off-target next generation sequencing data from 883 individuals with genetically unsolved HI. Due to the limitations of off-target copy number variant (CNV) calling the CNV boundaries are only accurate to ±200kb. To ensure that the novel deletions identified in our cohort were rare in the population we screened 6574 in-house controls for deletions in the same region using the same method. We then screened for deletions in this region in two population control cohorts: UK Biobank (n = 488,377) [18] and the gnomAD structural variant (SV) database v2.1 (n = 150,119) [19].

### Confirmation of deletions

Deletions identified from off-target CNV calling were confirmed by an independent cytogenetics analysis (patient 4) or by digital droplet PCR (ddPCR) (patient 5). ddPCR (Bio-Rad QX200 system) involved an EvaGreen dye-binding assay to measure dosage at 11 target sites across a 6.6 Mb region (Chr20:17930867-24565591) which extended across the *FOXA2* gene [20]. Targets and primer sequences are provided in Supplementary Table 1. This analysis also allowed for refinement of the 5’ breakpoint in patient 5.

When a deletion was identified, parental samples were tested by ddPCR as described above (n = 3 families) or by off-target CNV calling from targeted panel data (n = 2 families) [17]. Family relationships were confirmed by microsatellite analysis (PowerPlex kit, Promega, Southampton, UK).

### Interrogation of genome sequencing data and epigenomic data to decipher disease mechanism

Genome sequencing data was available for three probands. This was analysed to search for a recessive variant unmasked by the deletion. To do this we called all non-synonymous variants using an approach based on the GATK best practice guidelines. Briefly, variants were called using GATK haplotype caller and annotated using Alamut Batch (Interactive Biosoftware v1.11, Rouen, France). All variants common in gnomAD 2.1.1 (AC > 500) were excluded.

In a further attempt to pinpoint the genomic region causative of the HI we next searched for de novo variants within the minimal deleted region in 103 genetically unsolved individuals with HI where genome sequencing data was available on the proband and their unaffected parents. Variants were called using the pipeline outlined above then confirmed as de novo by DeNovoCNN [21]. This analysis included point mutations and deletions >2 kb.

#### Analysis of gene expression

To assess the expression of genes disrupted by the novel deletion we studied publicly available human islet single-cell RNA-seq (scRNA-seq) datasets collected over a time course of pancreatic differentiation projected onto a differentiation pseudotime obtained from [22]. We identified consistent temporal trends using Gaussian Process regression, following the approach that we have previously applied [12]. For scRNA-seq data in human islets [23], accession GSE101207, we used gene counts per cell for the size healthy donors and normalized by depth per cell. All accessions used in this analysis are provided in Supplementary Table 2.

#### Epigenomic analysis

We next interrogated assay for transposase-accessible chromatin sequencing (ATAC-seq) datasets to identify whether the loss of minimal deleted region has the potential to impact the regulation of *FOXA2* expression. Quantification of genomic single-nucleus ATAC-seq (snATAC-seq), bulk ATAC-seq and chromatin immunoprecipitation followed by sequencing (ChIP-seq) data was performed as in Wakeling et al. [12]. For human islet snATAC-seq data from Chiou et al. [24] (GSE160472), total snATAC-seq beta-cell peaks were obtained by generating a bam file of all reads assigned to beta_1 cluster (obtained https://github.com/kjgaulton/pipelines/tree/master/islet_snATAC_pipeline), and then calling peaks with MACS2 v2.2.7.1.

To determine the number of distinct active regulatory regions within the *FOXA2* control region, we calculated depth normalised transcription factor occupancy/chromatin accessibility in reads per million of single-end fragments extended 120 bp and the paired-end fragments of all islet transcription factor binding data and snATAC data for alpha_1 and beta_1 clusters. We then took the union of all intervals for which at least one dataset exceeded 1.5 RPM. Human islet Hi-C data were obtained from experiment accession TSTSR043623 and file accession DFF064KIG (.hic file) and TSTFF938730 (bedpe file) [25], were processed and visualised as in [12]. EndoC-βH1 RNA Pol II ChIA-PET enhancer promoter loops [26] were obtained from GSM3333915.

## Results

### Overlapping deletions on 20p11.2 in five individuals

Using whole genome sequencing we identified large overlapping deletions on chromosome 20p11.2 in three unrelated probands with HI. No further large heterozygous or homozygous deletions were detected in three or more individuals.

Using off-target sequencing data generated from routine testing we next screened for deletions which overlapped this region in 883 individuals with genetically unsolved HI. This analysis identified overlapping deletions in two additional probands.

Testing of parental samples confirmed that the deletions had arisen de novo in four individuals whilst one child (patient 3) had inherited the variant from their unaffected mother, who was also heterozygous. Biochemistry studies had not been performed on the mother to investigate hyperinsulinism and pituitary function and samples from the maternal grandparents were not available for co-segregation studies.

**Fig. 1:**
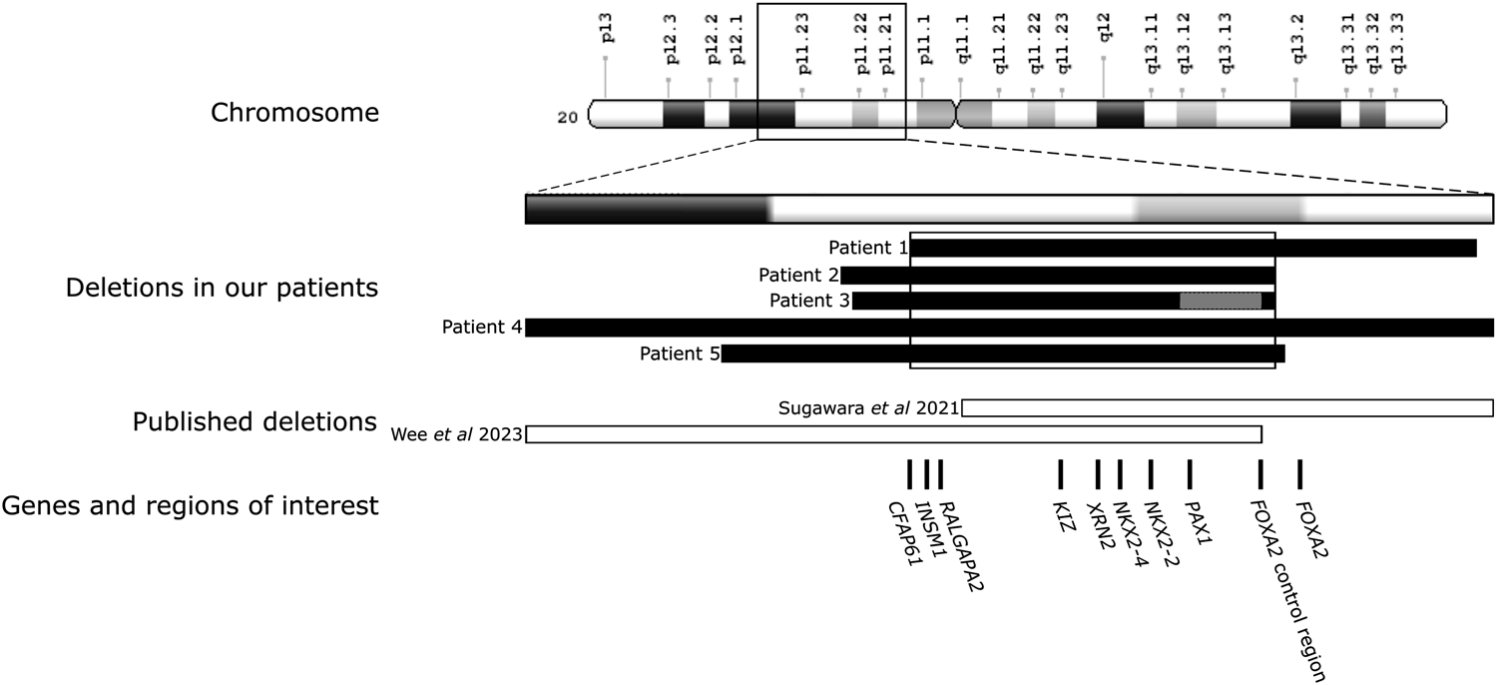
A diagram showing the 20p11.2 deletions in the five probands identified in our study and two published cases [36, 39] with hyperinsulinism who had deletions that overlapped those in our probands. The chromosomal location of the deletions is at the top. The deletions are depicted by bars—deletions identified in our patients in black, published deletions unfilled. A grey bar represents the inversion in the middle of two deletions that was identified in patient 3. The box shows the minimal deleted region shared between our five probands. The approximate positions of the genes and the putative *FOXA2* control region (Chr20:22,359,758-22,516,969) are marked on the diagram.

All five deletions encompassed a non-imprinted region on chromosome 20p11.2. Patient 3 had a complex variant with a deletion of 2.1 Mb (Chr20:g.19507014-21588883del) followed by a 0.9 Mb inversion (Chr20:g.21,588,883-22,510,428inv) and a further 15Kb deletion (Chr20:g.22,510,428-22,525,896del). The deletions in the five patients ranged from 3 to 8 Mb and had a minimal shared overlap of 2.4Mbp. This was fine mapped to 2,367,250 bp using the genome sequencing data from three individuals (Chr20:20,158,646-22,525,896) (Fig. 1). No large deletions spanning the minimal deleted region were identified in >7000 internal controls or >600,000 population controls recruited to the UK Biobank and gnomAD SV.

The ~2.4 Mb shared minimal deleted region contains the entire coding region of seven genes (*INSM1*, *RALGAPA2*, *KIZ*, *XRN2*, *NKX2-4*, *NKX2-2* and *PAX1*). The 5’ boundary dissected *CFAP61*, removing 14 of its 27 exons and the 3’ boundary was located 37 kb downstream of the coding region of *FOXA2* (Fig. 1). In two individuals the deletion extended over the entire *FOXA2* coding sequence.

To test whether the deletions were unmasking a recessive variant we analysed the genome sequencing data that was available from three individuals (patients 1–3). No rare non-synonymous variants in the coding regions of the genes within the minimal deleted region (*CFAP61*, *INSM1*, *RALGAPA2*, *KIZ*, *XRN2*, *NKX2-4*, *NKX2-2* and *PAX1*) or rare non-coding variants shared between the three patients were identified. We also searched for de novo variants in genome sequencing data from a further 103 individuals with genetically unsolved HI to see if we could pinpoint the disease-causing gene or regulatory region. No de novo non-synonymous variants were detected and no non-coding de novo variants within 10 kb of each other were found, making them unlikely to be within the same regulatory region.

### 20p11.2 deletions cause HI and extra-pancreatic features

The five probands with a 20p11.2 deletion were diagnosed with HI between the ages of 1 day and 52 weeks (Supplementary table 3). All individuals were treated with diazoxide. Three individuals continued drug treatment at a median age of 4 years (range 3–12 years), while HI remitted at 6 months in one child (Patient 5), and one child showed poor response to treatment necessitating a near-total pancreatectomy at the age of 3.3 years (Patient 1). Pancreatic tissue had not been stored following surgery.

Extra-pancreatic features were observed in all five individuals. Patient 1, whose deletion included the coding region of *FOXA2* had dysmorphic features, mild motor delay, and hypoplastic anterior pituitary gland with other midline defects. They had recently been diagnosed with growth hormone (GH) deficiency at the age of 7 years. The second child (Patient 4), whose deletion included the *FOXA2* coding region, had ventricular septal defect and horseshoe kidney, but no dysmorphic features or concerns with pituitary function or development by the age of 4 years. The three individuals (Patients 2, 3 and 5) whose deletion did not extend over the coding region of *FOXA2*, had not been identified with pituitary hormone deficiencies by the median age of 7 years (range 3–12) but had subtle facial features and developmental delay. Additional features were reported in two of these patients, including mild ventricular hypertrophy in infancy (possibly due to the HI) and epilepsy in Patient 2, and resolved patent ductus artery and anal stenosis in Patient 5.

### Epigenomic analysis of the minimal deleted region

To investigate the functional impact of the deletions, we assessed the expression of *FOXA2* and the eight genes within the minimal deleted region whose coding sequence was partially or fully lost. Human islet single-cell RNA-seq data [23] demonstrated that all genes except for *CFAP61*, *NKX2-4*, and *PAX1* are expressed in islets and pancreatic beta-cells. *FOXA2*, *NKX2-2*, *XRN2, INSM1* were most highly expressed (Supplementary Fig. 1).

**Fig. 2:**
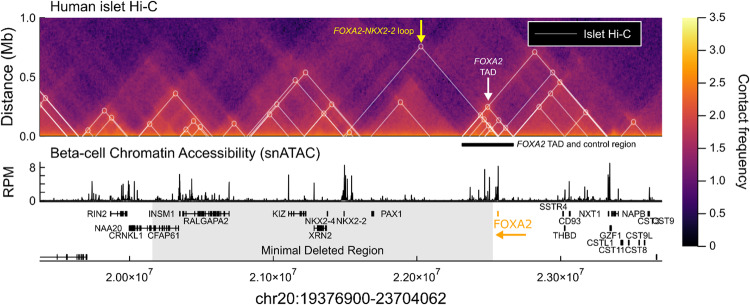
Regulatory landscape of the Chr20 minimal deleted region. Human islet Hi-C contact frequency heatmap [25] (top), and single-nucleus ATAC-seq beta-cell chromatin accessibility [24] (bottom). White triangles and circles mark chromatin loops called in [25]. The white arrow and black bar mark the topologically associated domain (TAD) spanning *FOXA2* and minimal deleted region. The yellow arrow highlights the chromatin loop between *FOXA2* and *NKX2-2*. The grey region marks the minimal deleted region. *FOXA2* marked in orange with an arrow indicating direction of transcription.

Motivated by the knowledge that heterozygous loss-of-function variants in *FOXA2* cause HI [27–30] and that the deletion in two of the individuals encompassed the entire *FOXA2* gene, we next assessed the gene regulatory potential of the minimal deleted region. Human islet single-nuclei ATAC-seq [24] revealed 149 distinct regions of chromatin accessibility in insulin-secreting beta-cells, including the promoters of the five beta-cell expressed genes (*INSM1*, *RALGAPA2*, *KIZ*, *XRN2* and *NKX2-2*) (Fig. 2). We next determined whether any of these regulatory regions had the potential to regulate genes outside the minimal deleted region by examining chromatin conformation by human islet Hi-C data [25]. Amongst multiple three-dimensional contacts, we found a ~ 350 kb topologically associated domain spanning *FOXA2* and the minimal deleted region (Fig. 2, white arrow). This region has previously been reported to contain islet super enhancers [31–33].

We found multiple lines of evidence to suggest that this region acts as a *FOXA2* control region that regulates expression across developing and adult tissues. We identified a 220 kb region (Chr20:22,359,758-22,516,969) encompassing 77 distinct regulatory regions in islets (Supplementary Fig. 2). We found that these regions were marked with active enhancer mark histone 3 lysine 27 acetylation (H3K27ac), and moreover, that the activity of these regulatory regions varied across pancreatic cell differentiation, between pancreas and liver where *FOXA2* is also highly expressed, and within islet cell types (Supplementary Fig. 2). For the latter, the regulatory region most strongly bound by the beta-cell restricted transcription factor PDX1 is accessible in beta-cells and not alpha-cells, suggesting this region confers beta-cell specific control of FOXA2.

Beyond the *FOXA2* control region, human islet chromatin conformation data reveals a CTCF-CTCF loop connecting the promoters of *NKX2-2* and *FOXA2* identifying a co-regulatory mechanism between these two genes that is lost in the minimal deleted region (Fig. 2, yellow arrow). It should be noted that *FOXA2* and *NKX2-2* occupy many of the same regulatory loci, with *FOXA2* sharing 45% of its human islet binding sites with *NKX2-*2 [31], and therefore a loss of *NKX2-2* may impact *FOXA2* binding at loci where there is a dependency between these factors.

Together, our analysis of public epigenomic data indicates that in addition to the loss of coding sequence of eight genes, the minimal deleted region includes multiple regulatory elements whose loss is predicted to disrupt *FOXA2* expression.

## Discussion

We have identified large overlapping deletions on chromosome 20p11.2 in five individuals with HI. In four, the deletions had arisen de novo providing strong evidence for pathogenicity, while in one proband a complex deletion/inversion variant was identified which had been inherited from their unaffected mother, in keeping with variable penetrance. Variable penetrance has been well reported in other dominantly-inherited forms of HI including *ABCC8* [34] and *HNF4A* [35]. The absence of rare single-nucleotide variants on the non-deleted allele in three individuals and the lack of an imprinted region suggests that these deletions are causing disease through haploinsufficiency.

Large proximal chromosome 20p11 deletions of varying size are rare and have been associated with developmental and structural abnormalities of varying severity [30, 36–40]. While many of these features are non-specific, most individuals with 20p11 deletions encompassing *FOXA2*, as well as those with HI due to pathogenic missense variants in *FOXA2*, have presented with (pan)hypopituitarism due to pituitary gland defect, midline defects affecting abdominal or cardiovascular organs and the central nervous system, developmental delay and dysmorphism [30, 36–40]. All five individuals in this study had varying syndromic features which overlap with previously described cases with proximal 20p deletions.

For the two individuals whose deletions included the coding region of *FOXA2*, Patient 1 showed clear midline and pituitary defects resulting in GH deficiency and Patient 4 had two structural abnormalities which could be considered as midline defects. Follow-up is though needed for this patient as they are currently only 4 years of age, which might be too young to have presented with clear signs of pituitary hormone deficiencies.

All five probands in this study were referred for genetic testing for HI, a condition not commonly associated with 20p11deletions. A literature search identified only two cases with HI caused by heterozygous 20p11 deletions, both had GH deficiency. One was a 5.8Mbp deletion on 20p11.22-p11.21 [39] that covers almost the entirety of our minimal deleted region including part of the *FOXA2* control region we identified. The other is a 2.48Mbp deletion on chromosome 20p11.23-p11.21 [36], that overlaps with our minimal deleted region but does not extend over *CFAP61*, *INSM1* or *RALGAPA2* suggesting that the disruption of these genes is not responsible for the HI (see Fig. 1 for a visual representation).

Considering the overlapping deletion reported by Sugawara et al. [36] we are left with five genes within the minimal deleted region for HI: *KIZ*, *XRN2*, *NKX2-4*, *NKX2-2* and *PAX1*. Convincing data to support a role for each of these genes in the aetiology of HI is lacking. *XRN2*, *NKX2-4*, *NKX2-2* and *PAX1* are tolerant to truncating variants based on their gnomAD pLI score (threshold pLI >= 0.9) (data unavailable for *KIZ*) [41] and scRNA-seq data shows that *NKX2-4*, and *PAX1* are not expressed in islets and insulin-producing pancreatic beta-cells. Only *NKX2-2* is known to have a role within the pancreas where it encodes a transcription factor involved in pancreatic cell differentiation, maintenance of beta-cell function and formation of islet structure [42]. Importantly however, bi-allelic null variants in *NKX2-2* cause neonatal diabetes and the parents, who are heterozygous carriers, do not have hypoglycaemia [43]. *NKX2-2* expression has also been shown to be increased rather than decreased in islets from patients with HI [44].

*FOXA2* in contrast is a strong candidate gene for the HI despite the coding sequence of the gene only being deleted in two of the five individuals in our study and one of the two individuals previously reported to have HI caused by deletions on chromosome 20p [36, 39]. This gene encodes a transcription factor that has an essential role in pancreatic development [45]. In mature pancreatic islet cells, FOXA2 regulates the expression of genes that encode key components of the insulin secretion pathway [46, 47]. Most convincingly, heterozygous loss-of-function coding variants in *FOXA2* have been reported in at least seven individuals with hypoglycaemia, most of whom also had confirmed HI [27–30, 40, 48, 49]. Interestingly Kaygusuz et al. [30] reported a patient with an 8.53 Mb deletion that included *FOXA2* who presented with diabetes and the patient with transient hypoglycaemia reported by Stekelenburg et al. [48] developed diabetes later in childhood, suggesting there could be a more variable pancreatic phenotype caused by variants in *FOXA2*.

In keeping with a role for *FOXA2* in the aetiology of HI in our three patients who have the coding region of the gene intact, our analysis of functional genomic data highlighted a *FOXA2* control region that was deleted in all individuals. This control region has strong three-dimensional contact with the *FOXA2* promoter and comprises at least 77 distinct active regulatory regions within human islets. We find that these regulatory regions exhibit cell-specific regulatory activity, varying in activity across differentiation and adult tissues where *FOXA2* is expressed; it therefore likely plays a role in fine-tuning *FOXA2* expression. Part of this *FOXA2* control region was also deleted in the patient with HI studied by Wee et al. [39]. Therefore, all seven patients (five in this study and two from the literature) with a deletion on chromosome 20p11.2 and HI have a deletion that affects the *FOXA2* control region. This deletion extends over the *FOXA2* coding sequence in three cases. Follow-up of all these patients is therefore warranted to examine whether they go on to develop similar features to those reported in individuals with *FOXA2* coding variants, particularly (pan)hypopituitarism.

We acknowledge that our patients have deletions affecting significant components of the islet transcription factor network and the phenotypes and impact on gene expression could be multifaceted. Given that *FOXA2* and *NKX2-2* bind many of the same regulatory loci, with *FOXA2* sharing 45% of its human islet binding sites with *NKX2-*2 [31], it remains possible that *FOXA2* binding is altered at sites it shares with *NKX2-2* (such a dependency has been shown for *PDX1*, another *FOXA2* co-binding factor [50]). We therefore cannot exclude that concomitant loss of *NKX2-2* with loss of *FOXA2* regulatory control is necessary for HI to develop in these individuals. Furthermore, no de novo coding variants or clusters of non-coding variants within the minimal deleted region were identified in a large unrelated cohort of individuals with genetically unsolved HI. This suggests that single nucleotide variants within this region are an extremely rare cause of HI or that large structural variants, that disrupt multiple genes/regulatory regions, are required to cause disease. Identifying further deletions or disease-causing single nucleotide variants that refine the critical region will be important to gain knowledge of the precise molecular mechanism(s) of HI.

In conclusion, we have identified a 2.4 Mb deletion on chromosome 20p11.2 as a cause of syndromic HI in five individuals and we recommend that this chromosome region is included by genomic laboratories in their screening panels for this condition, especially when syndromic disease is suspected. Our findings suggest that these deletions cause HI through the disruption of *FOXA2*, either by removing its entire coding region or by disrupting non-coding regulatory elements that are critical for controlling *FOXA2* expression within the pancreatic beta-cell. These findings further highlight the critical role of studying large structural variants to gain insights into non-coding gene regulation and to aid discovery of novel causes of Mendelian disease.

### Supplementary information

Supplemental material

## Data Availability

All non-clinical data analyzed during this study are included in this article (and its Supplementary Information). The 20p11.2 variants reported in this study were uploaded to ClinVar (SUB14235415). Clinical and genotype data can be used to identify individuals and are therefore available only through collaboration to experienced teams working on approved studies examining the mechanisms, cause, diagnosis and treatment of diabetes and other beta cell disorders. Requests for collaboration will be considered by a steering committee following an application to the Genetic Beta Cell Research Bank (https://www.diabetesgenes.org/current-research/genetic-beta-cell-research-bank/). Contact by email should be directed to S. Flanagan (s.flanagan@exeter.ac.uk). All requests for access to data will be responded to within 14 d. Accession codes and DOI numbers for all ChIP-seq, ATAC-seq, RNA-seq and scRNA-seq datasets are provided in Supplementary Table 2. We used the Genome Reference Consortium Human Build 37 (GRCh37) to annotate genetic data (accession number GCF_000001405.13). Details of this assembly are provided at https://www.ncbi.nlm.nih.gov/assembly/GCF_000001405.13/.
