# Supplementary material for: Chromosome 20p11.2 deletions cause congenital hyperinsulinism via the loss of FOXA2 or its regulatory elements
Source: Eur J Hum Genet. 2024 Apr 11;32(7):813–8. doi: 10.1038/s41431-024-01593-z (PMC11220097; doi:10.1038/s41431-024-01593-z)
Supplement: Supplementary file 1 — Supplemental material [file 41431_2024_1593_MOESM1_ESM.docx]

**Supplementary Information**

**Supplementary Table 1:** Genomic targets and primers for the droplet digital PCR testing of the 20p.11.2 deletions.

| **GRCh37 genomic coordinates** | **Forward** | **Reverse** |
| --- | --- | --- |
| chr20:17930867-17930972 | TTGTGTGAAAGCTGACAAAATG | CAAGTCTTTTTATCCAAACACAGC |
| chr20:18794590-18794685 | GCTTCCCAGTGTTTCAGGAC | TGGTGATGTCCAGCTGAAAG |
| chr20:19956252-19956341 | ACTTCGGGTGCTTAGTGCAG | GGATGGTCTGCAGCATGTC |
| chr20:21687223-21687326 | GCTGCGTGAGCAAGATCC | CTTGACCACGTTGGGAGTG |
| chr20:22262921-22263030 | AAGGACATGAATGACAAGATC | GAGCTTGATGGAACAATCACC |
| chr20:22381210-22381300 | TTTGCTCCTTTTGACTGCTG | ACCCATGTGTTCATCCACTG |
| chr20:22441027-22441122 | TGAGTGGCAAACACCTGAAC | CAAGGCCTCTTGAGGTATGC |
| chr20:22550245-22550351 | GCCTGGAAATTTGTCTGAGC | TTGCAATGTCTGTGCAGGTC |
| chr20:22564830-22564927 | TTTAAACTGCCATGCACTCG | CTCGGGCTCTGCATAGTAGC |
| chr20:23370596-23370687 | GCTTTGGTTCTTGATTTCAGC | TCAATGGCTTTCTGGTACTGC |
| chr20:24565487-24565591 | TGCAGAGCGACTACTCAAGC | AGAAGCAGCAGAGCATGGAG |

**Supplemental Table 2:** Table describing all public genomic datasets used in this study.

ES – embryonic stem, DE – definitive endoderm, GT – gut tube, PP – pancreatic progenitor.

| Accession | DOI | Description | Cell | Target |
| --- | --- | --- | --- | --- |
| GSE149148 | doi.org/10.7554/eLife.59067 | ATAC-seq, ChIP-seq for TFs over pancreatic differentiation | ES, DE, GT, PP1, PP2 | FOXA2, ATAC |
| E-MTAB-1919 | doi.org/10.1038/ng.2870 | ChIP-seq for TFs in islets | Islets | CTCF, FOXA2, H2AZ, H3K27ac, MAFB, NKX2_2, NKX6_1, PDX1 |
| E-MTAB-1990,  E-MTAB-3061 | doi.org/10.1038/ncb3160 | ChIP-seq for TFs liver buds | LiverBud, | FOXA2 |
| GSE148368 | doi.org/10.1038/s41467-021-26950-0 | ChIP-seq for TFs in pancreatic and liver differentiation | GT, HP | FOXA2 |
| GSE160472 | doi.org/10.1038/s41588-021-00823-0 | Single nuclei ATAC-seq in islets | Islets | ATAC |
| GSE101207 | doi.org/10.1016/j.celrep.2019.02.043 | Single cell RNA-seq in islets | Islets |  |
| GSE143783 | doi.org/10.1038/s42255-020-00314-2 | Single cell RNA-seq of beta-like cell differentiation, abundances projected on pseuodotime available in ref's Supplementary Table 4 | ES cell to beta-like cell differentiation |  |

**Supplementary Table 3:** Summary of clinical features and genetic findings in individuals with 20p11.2 deletions.

Genetic coordinates relate to GRCh37. * denotes the total genomic region disrupted in this patient which includes an inverted region (details provided in the main text). ACTH, Adrenocorticotropic hormone; BOHB, Beta-hydroxybutyrate; EEG, Electroencephalogram; FFA, Free fatty acids; FSH, Follicle stimulating hormone; FT4, Free thyroxine (T4); GA, Gestational age; GH, Growth hormone; HI, Hyperinsulinism; IGF-1, Insulin-like growth factor 1; IGFBP3, Insulin-like growth factor binding protein 3; LH, Luteinizing hormone; MRI, Magnetic resonance imaging; NA, Not available; PRL, Prolactin; T3, Triiodothyronine; TSH, Thyroid stimulation hormone; ↔, at average range; ↑, above average range; ↓, below average range

|  | **Patient 1** | **Patient 2** | **Patient 3** | **Patient 4** | **Patient 5** |
| --- | --- | --- | --- | --- | --- |
| **Coordinates of 20p11.2 deletions** | Chr20:20158646– 24080787 | Chr20:19434987–22528253 | Chr20:19507014–22525896* | Chr20:16400000–24400000 | Ch20:18200000–22600000 |
| **Del includes *FOXA2*** | Yes | No | No | Yes | No |
| **Sex** | Male | Female | Female | Female | Male |
| **Age at latest follow-up** | 7 years | 12 years | 3 years | 4 years | 7 years |
| **Birth weight (centile), GA** | 4.2 kg (99^th^)  38 weeks | 3.7kg (63^rd^)  41 weeks | 4.5 kg (99^th^)  40 weeks | 2.8 kg (15^th^)  39 weeks | 4.0 kg (73^rd^)  40 weeks |
| **Age at Presentation of HI** | Hypoglycemia at birth resolved, HI at 6 months | 12 weeks | 52 weeks | 2 days | 1 day |
| **Critical sample during hypoglycemia** | | | | | |
| **Glucose, mmol/L (Insulin, pmol/L)** | 2.3  (117) | 2.3  (582) | 3.0  (14) | <0.3  (209) | 1.4  (53) |
| **Other biochemistry during hypoglycemia** | NA | BOHB 0.26 nmol/L ↓  FFA 0.34 mmol/L ↓ Cortisol 684 nmol/L ↑  GH 16.7 µg/L ↔ | NA | BOHB 0.33mmol/L ↓  FFA 0.98mmol/l ↓  GH 1.2ug/l ↓ | BOHB <0.05 mmol/L ↓  FFA 0.10 mmol/L ↓  Cortisol 879 nmol/L ↑  GH 38.2µg/L ↑ |
| **Treatment for HI** | | | | | |
| **Diazoxide-responsive**  **(initial dose)** | No (11 mg/kg/d)  80% pancreatectomy aged 3.3 years,  histology of diffuse HI | Yes  (10 mg/kg/d) | Yes  (10 mg/kg/d) | Yes  (9mg/kg/d) | Yes  (4.2 mg/kg/d) |
| **Current treatment** | Frequent feeds | Diazoxide  (6.2mg/kg/d) | Diazoxide  (6mg/kg/d) | Diazoxide  (7mg/kg/d) | None - diazoxide stopped at 6 months |
| **Other features** | | | | | |
| **Pituitary hormone examination**  **(normal range)** | GH deficiency diagnosed at 7 years of age (on GH treatment.  At 7 years of age  Cortisol ↔  TSH ↔  FT4 ↔  Brain MRI: Hypoplastic AP and ectopic PP, Chiari 1 malformation.  At 4 years of age  TSH 2.3 mU/L ↔  (0.49–4.9)  FT4 8.7 pmol/L ↔  (6.1–12.9)  IGF-1 33 ng/mL ↔  (22–208) | No pituitary dysfunction identified.  At 7 years of age  No growth delay (height 75^th^ centile). No pubertal delay.  TSH 2.15 mU/L ↔  (0.1–5.5)  FT4 17.8 pmol/L ↔  (10–22)  Brain MRI: Normal | No pituitary dysfunction identified.  At 3 years of age  TSH 0.74 mUI/ml ↔  (0.27-4.2)  FT4 16.34 pmol/L  (11.58-12.88)  T3 1.63 ng/ml ↔  (0.8-2.0)  Cortisol 248 nmol/L ↔  (132-530)  ACTH 3.3 pmol/L ↔  (0-10.12)  PRL 15.09 mcg/L ↔  (4.79 –23.3)  IGF-1 63.6 ng/ml ↔  (29-118)  IGFBP3 2.16 ug/ml ↔  (0.9-4.7)  Height 10–25^th^ centile (appropriate for parental heights) | No pituitary dysfunction identfied. Pituitary MRI and GH stimulation test planned.  At 4 years of age  TSH 2.54 mU/L ↔  (0.7–4.17)  FT4 11.5 pmol/L ↔  (10.3–17)  IGF-1 6.7 nmol/L ↔  (6–24)  Height at 2^nd^ centile  (–1.9 SDS)  At 2 years of age  IGF-1 6.3 nmol/L ↔  (3–17)  IGFBP3 1.9 mg/L ↔  (1.2–3.7)  At 2 months of age  Cortisol (morning) 406 nmol/L ↔ | No pituitary dysfunction identified.  At 7 years of age  TSH 1.49 mU/L ↔  (0.50–3.80)  FT4 15.9 pmol/L ↔  (10.8–22.9)  IGF-1 31.5 nmol/L ↑  (7.1–26.8)  Height at 75^th^ centile.  At 4.5 years of age  LH <0.1 U/L ↔  (<2.6)  FSH 0.3 U/L ↔  (0.2–3.0)  PRL 465 mU/L ↔  (82–967)  At birth  GH 12.9 ug/L ↔  Cortisol 111 nmol/L ↔ |
| **Structural defects** | Cryptorchidism, micro-penis, left lacrimal duct stenosis, inguinal hernia | Mild left ventricular hypertrophy in infancy  (HI-related, resolved) | None | Horseshoe Kidney, ventricular septal defect | Anal stenosis, patent ductus artery (resolved) |
| **Dysmorphology** | Right preauricular pit, telecantus, wide & flat nasal bridge, clinodactyly (3^rd^ toe), uneven folds between upper legs | Long eyelashes, synophrysis, and mildly dysmorphic facial features (possibly diazoxide-related) | Subtle features: long face, epicanthus, eversion of lower eyelid, retro-micrognathia, thin lips, short columella | None | Hypertelorism |
| **Development and neurology** | Mild motor delay | Speech delay, mild motor delay, mild learning difficulties, epilepsy (focal features on EEG) | Developmental delay | No concerns with development | Developmental delay and learning difficulties |


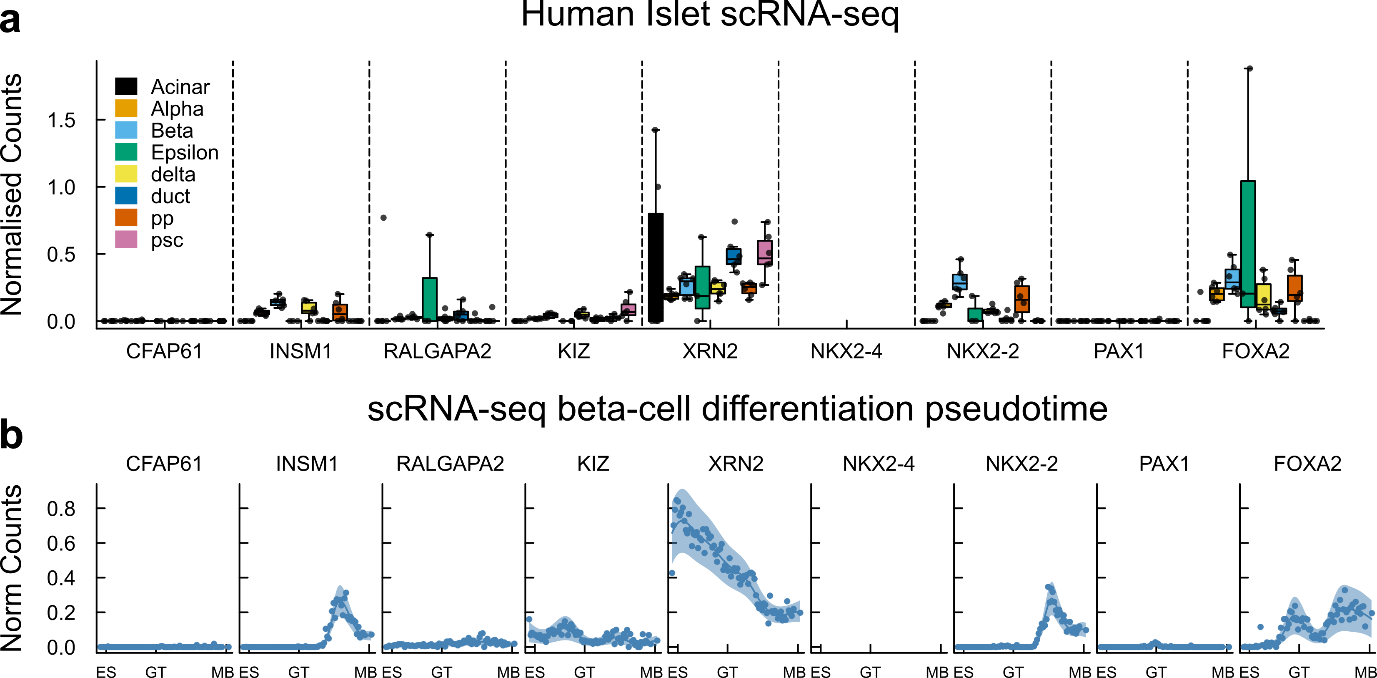


**Supplementary Figure 1:** Expression of genes partially or fully deleted by minimal deleted region and FOXA2.

**a.** Human islet single-cell RNA-seq (scRNA-seq) (GSE101207 [1]) expression data of the 8 genes whose sequence is disrupted and FOXA2 shown for reference. Data points give mean normalised counts of 6 independent donors for scRNA-seq cell type clusters defined in original publication (pp – pancreatic polypeptide, psc= pancreatic stellate cell). Boxplot central lines give the median, boxes span interquartile range, and whiskers extend to the furthest point within 1.5x IQR of the box. Order of cell types (left to right) matches order of legend (top to bottom).

**b.** scRNA-seq expression over pancreatic cell differentiation from embryonic stem (ES) cells to maturing beta-cells (MB), mapped onto a beta-cell differentiation pseudotime [2] that includes gut tube stage (GT). scRNA-seq data-points on pseudotime shown with Gaussian process regression, line and shaded region mark posterior Gaussian process median and 95% confidence intervals.


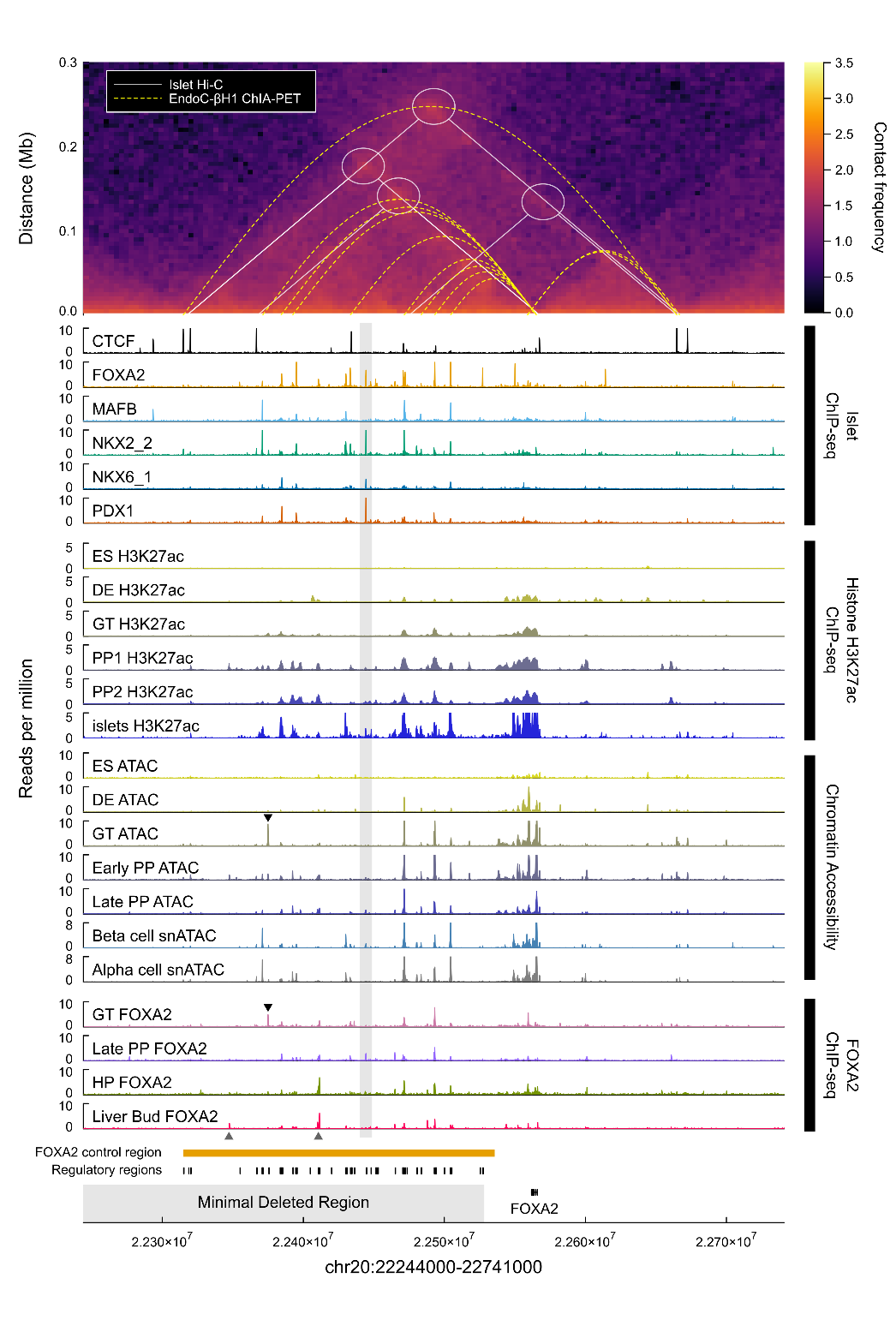


**Supplementary Figure 2:** Regulatory activity of FOXA2 control region.

Human islet Hi-C contact frequencies, transcription factor binding in multiple cell types and chromatin accessibility show regulatory activity of FOXA2 control region (orange bar).

Top: Heatmap gives contact frequencies of human islet Hi-C (5 kb bins) [3], white lines and circles describe CTCF-CTCF chromatin loops called in same study. Yellow dotted lines mark EndoC-βH1 RNA Pol II ChIA-PET enhancer-promoter loops (GSM3333915 [4]), highlighting contact between individual transcription factor binding sites and *FOXA2* promoter.

Bottom: human islet chromatin immunoprecipitation followed by sequencing (ChIP-seq) data [5]; H3K27ac ChIP-seq over pancreatic cell differentiation [6] and in human islets [5], ES – embryonic stem, DE – definitive endoderm, GT – gut tube, PP – pancreatic progenitor; chromatin accessibility data over pancreatic cell differentiation [6]; single nuclei assay for transposase-accessible chromatin sequencing (snATAC) of human islets showing alpha and beta cell clusters [7], (beta_1 and alpha_1 clusters from original study shown); FOXA2 ChIP-seq from *in vitro* differentiation gut tube (GT), pancreatic progenitors (PP), and hepatic progenitors (HP) [8], and *in vivo* in liver bud [9].

Individual regulatory regions shown strong contact with FOXA2 promoter by Hi-C and RNA Pol II ChIA-PET, activity of regulatory regions varies by factor and cell type. Beta-cell specific regulatory region, marked by grey vertical bar. FOXA2 ChIP-seq shows varied binding of FOXA2 at its own control region across differentiation and between pancreas and liver. Black down triangle (▼) marks gut-tube specific regulatory region by chromatin accessibility and FOXA2 binding. Grey up triangle (▲) marks liver dominant binding of FOXA2.

**Supplementary references**

1. Fang, Z., et al., *Single-Cell Heterogeneity Analysis and CRISPR Screen Identify Key β-Cell-Specific Disease Genes.* Cell Rep, 2019. **26**(11): p. 3132-3144.e7.

2. Weng, C., et al., *Single-cell lineage analysis reveals extensive multimodal transcriptional control during directed beta-cell differentiation.* Nat Metab, 2020. **2**(12): p. 1443-1458.

3. Greenwald, W.W., et al., *Pancreatic islet chromatin accessibility and conformation reveals distal enhancer networks of type 2 diabetes risk.* Nat Commun, 2019. **10**(1): p. 2078.

4. Lawlor, N., et al., *Multiomic Profiling Identifies cis-Regulatory Networks Underlying Human Pancreatic β Cell Identity and Function.* Cell Rep, 2019. **26**(3): p. 788-801.e6.

5. Pasquali, L., et al., *Pancreatic islet enhancer clusters enriched in type 2 diabetes risk-associated variants.* Nat Genet, 2014. **46**(2): p. 136-143.

6. Geusz, R.J., et al., *Pancreatic progenitor epigenome maps prioritize type 2 diabetes risk genes with roles in development.* Elife, 2021. **10**.

7. Chiou, J., et al., *Single-cell chromatin accessibility identifies pancreatic islet cell type- and state-specific regulatory programs of diabetes risk.* Nat Genet, 2021. **53**(4): p. 455-466.

8. Geusz, R.J., et al., *Sequence logic at enhancers governs a dual mechanism of endodermal organ fate induction by FOXA pioneer factors.* Nat Commun, 2021. **12**(1): p. 6636.

9. Cebola, I., et al., *TEAD and YAP regulate the enhancer network of human embryonic pancreatic progenitors.* Nat Cell Biol, 2015. **17**(5): p. 615-626.
